# Experimental and computational study on roles of WO_x_ promoting strong metal support promoter interaction in Pt catalysts during glycerol hydrogenolysis

**DOI:** 10.1038/s41598-020-79764-3

**Published:** 2021-01-12

**Authors:** Tinnakorn Saelee, Poonnapa Limsoonthakul, Phakaorn Aphichoksiri, Meena Rittiruam, Mongkol Lerdpongsiripaisarn, Takanori Miyake, Hiromi Yamashita, Kohsuke Mori, Yasutaka Kuwahara, Supareak Praserthdam, Piyasan Praserthdam

**Affiliations:** 1 High-Performance Computing Unit (CECC-HCU), Centre of Excellence on Catalysis and Catalytic Reaction Engineering (CECC), Department of Chemical Engineering, Faculty of Engineering, Chulalongkorn University, Bangkok, 10330 Thailand; 2 Centre of Excellence on Catalysis and Catalytic Reaction Engineering (CECC), Department of Chemical Engineering, Faculty of Engineering, Chulalongkorn University, Bangkok, 10330 Thailand; 3 Faculty of Environmental and Urban Engineering Department of Chemical, Energy and Environmental Engineering, Kansai University, Suita, Osaka 564-8680 Japan; 4 Division of Materials and Manufacturing Science, Graduate School of Engineering, Osaka University, 1-1 Yamadaoka, Suita, Osaka 565-0871 Japan; 5 Saelee Research Group, Chulalongkorn University, Bangkok, 10330 Thailand; 6 Rittiruam Research Group, Chulalongkorn University, Bangkok, 10330 Thailand

**Keywords:** Chemical engineering, Electronic structure, Heterogeneous catalysis, Energy, Sustainability

## Abstract

Biodiesel is of high interest due to increased demand for energy with the concern regarding more sustainable production processes. However, an inevitable by-product is glycerol. Hence, the conversion of this by-product to higher-value chemicals, especially 1,3-propanediol (1,3-PDO) via glycerol hydrogenolysis reaction, is one of the most effective pathways towards a profitable process. In general, this process is catalyzed by a highly active Pt-based catalyst supported on γ-Al_2_O_3_. However, its low 1,3-PDO selectivity and stability due to surface deactivation of such catalysts remained. This led to the surface modification by WO_x_ to improve both the selectivity by means of the increased Brønsted acidity and the stability in terms of Pt leaching-resistance. Hence, we applied experimental and density functional theory (DFT)-based techniques to study the fundamentals of how WO_x_ modified the catalytic performance in the Pt/γ-Al_2_O_3_ catalyst and provided design guidelines. The effects of WO_x_ promoter on improved activity were due to the shifting of the total density of states towards the antibonding region evident by the total density of states (TDOS) profile. On the improved 1,3-PDO selectivity, the main reason was the increasing number of Brønsted acid sites due to the added WO_x_ promoter. Interestingly, the stability improvement was due to the strong metal-support interaction (SMSI) that occurred in the catalyst, like typical high leaching-resistant catalysts. Also, the observed strong metal-support-promoter interaction (SMSPI) is an additional effect preventing leaching. The SMSPI stemmed from additional bonding between the WO_x_ species and the Pt active site, which significantly strengthened Pt adsorption to support and a high electron transfer from both Pt and Al_2_O_3_ to WO_x_ promoter. This suggested that the promising promoter for our reaction performed in the liquid phase would improve the stability if SMSI occurred, where the special case of the WO_x_ promoter would even highly improve the stability through SMSPI. Nevertheless, various promoters that can promote SMSPI need investigations.

## Introduction

Due to the concern towards the depleting petroleum fuels and their impact on the environment, renewable resource-based fuels such as biodiesel have been continuously explored and utilized^1–9^. The biodiesel production process, although effective enough at present, still yielded a large number of by-products, especially glycerol, which is needed to be managed. The overstocked glycerol costed the biodiesel production process to become less efficient and profitable, raising a challenge towards a more sustainable process for biodiesel. One of the effective pathways is its conversion to higher-valued chemical feedstocks. Besides, the zero waste management for such an approach should be achieved through the catalyzed reaction of glycerol hydrogenolysis that produced value-added chemicals such as cyclic carbonate, which can be used in various applications^10–15^ such as a solvent-resistant coating and high modulus polymer. 1,2-Propanediol (1,2-PDO) is another candidate chemical as a precursor for ethylene glycol used as an anti-freezing fluid in food industries^14,16–18^. Also, another product of interest is 1,3-propanediol (1,3-PDO) used as the functional fluid in cosmetics, personal care, cleaning products^19–22^, and in polymer industries, where it was used as a monomer during the polytrimethylene terephthalate (PTT) polymerization^2,5,23–25^. Usually, 1,3-PDO is synthesized from petroleum-based feedstocks through hydrolysis of acrolein and hydroformylation of ethylene oxide. Thus, catalytic hydrogenolysis of glycerol to produce 1,3-PDO is a promising process.

The platinum (Pt) catalysts are highly active and selective; hence, a promising candidate for such a process as reported by Dasari et al*.*^26^, where up to 82.7% of 1,2-PDO selectivity at 34.6% of glycerol conversion was observed on an activated carbon-supported Pt catalyst. Also, Gandarias et al.^27^ investigated the use of the metal oxide support for a Pt-based catalyst on the same reaction and found that the amorphous silica-alumina supported Pt promoted the reaction via its high acidity. Therefore, to effectively design the Pt-based catalyst in this work, in which the Pt/γ-Al_2_O_3_ was used as a catalyst, the mechanism behind the reaction must be understood. It has been revealed that two reaction steps are involved in the reaction^9^. First, glycerol is dehydrated, forming acetol on the support prior to the hydrogenation of acetol to 1,2-PDO on the Pt site, where more acetol intermediates were produced on Pt than that on the support, indicating that the Pt site promotes dehydration of glycerol to acetol intermediate. However, the catalyst provided the low selectivity of 1,2-PDO and 1,3-PDO due to C–C bond cleavage forming by-products of ethylene glycol, acetone, formaldehyde, and ethanol. For such reasons, the modification of Pt-based catalyst by tungsten oxide doping (WO_x_) is of interest due to a significant improvement in selectivity towards 1,3-PDO. For instance, Kurosaka et al.^28^ reported ZrO_2_ support impregnated with WO_x_ and Pt as an active catalyst with a yield of 1,3-PDO around 24.2%, while García-Fernández et al*.*^29^ suggested the same results on the Pt/WO_x_/γ-Al_2_O_3_ catalyst, where high dispersion of polytungstate species on catalyst surface enhanced the selectivity up to 51.9% at 53.1% glycerol conversion. Although a combination of Pt and WO_x_ on γ-Al_2_O_3_ surface can improve the selectivity of 1,3-PDO beyond 1,2-PDO, the deactivations of a catalyst via leaching and agglomeration of active species are still difficult to avoid. In general, our catalyst having Pt and WO_x_ components experienced leaching and Pt agglomeration due to the liquid-phase environment, which promotes such deactivations^30–33^.

Hence, the understanding of the leaching and agglomeration of WO_x_-modified and unmodified Pt/γ-Al_2_O_3_ catalysts during the hydrogenolysis of glycerol is needed to better design the catalyst with higher 1,3-PDO selectivity and stability. The interaction of Pt with the support preventing agglomeration can be understood through the metal–support interaction. An appropriate metal–support interaction will lead to a catalyst surface with high resistance against metal active site leaching, where weak metal–support interaction (WMSI) and strong metal–support interaction (SMSI) were defined to describe the strength of the binding of the metal active site to the support^34^. In the case of hydrogenolysis, the SMSI is needed to prevent such a deactivation. Therefore, as evident from various works that the WO_x_ component helps improve the stability of the Pt-based catalyst, in this work, we provide an insight on how the WO_x_ performed in that improvement via experimental techniques coupled with density functional theory (DFT)-based analyses. From DFT analyses, the behavior of the electrons during the reaction described through charge transfer and electron density difference analysis can be used to illustrate the enhanced stability as also carried out in our previous works^31,35–37^.

## Results and discussion

### Chemical analysis

For fresh and reused catalysts, the Pt and W leaching from Pt/γ-Al_2_O_3_ and Pt/WO_x_/γ-Al_2_O_3_ catalysts during the hydrogenolysis of glycerol were confirmed via ICP-OES technique as summarized in Table 1, as well as the conversion of glycerol. For the activity and stability of a fresh Pt/γ-Al_2_O_3_ catalyst, 23.2% glycerol conversion and 7.2% Pt leaching were observed after the reaction. When such a catalyst was reused for the second round, the glycerol conversion reduced to 12.7%, with Pt leaching of 2.4%. This suggested that the loss of Pt active site through leaching played a significant role in the catalyst activity of glycerol hydrogenolysis.

**Table 1:** The amounts of Pt and W leaching from the surface of catalysts in the reaction solution based on the catalyst used.

Catalyst	Catalytic cycle	Glycerol conversion (%)	Leaching (%)	
			Pt	W
Pt/γ-Al_2_O_3_	Fresh	23.2	7.2	–
	Used	12.7	2.4^a^	–
Pt/WO_x_/γ-Al_2_O_3_	Fresh	35.8	0.2	30.3
	Used	30.0	0.0^a^	12.1^a^

^a^Leaching of used catalysts was calculated based on remained Pt and W on catalysts after the first hydrogenolysis reaction.

In the case when the WO_x_ promoter was added to the Pt/γ-Al_2_O_3_ catalyst forming Pt/WO_x_/γ-Al_2_O_3_, the fresh catalyst exhibited 35.8% glycerol conversion with Pt leaching of 0.2% and the W leaching of 30.3%. This addition of WO_x_ can be related directly to the prevention of Pt leaching. Moreover, for the used catalyst of the same system, glycerol conversion dropped slightly to 30.0%, and Pt leaching was not detected, whereas 12.1% of W leaching was found. This suggested that the known effect of SMSI between Pt and the support caused by the introduction of the WO_x_ component might play a role in a significant decrease in Pt leaching during hydrogenolysis. However, the strengthening of such interaction must be verified. As a result, at this point, the catalytic activity of glycerol hydrogenolysis related directly to the amount of Pt active sites. Hence, adding WO_x_ to Pt/γ-Al_2_O_3_ could improve catalytic activity by preventing Pt from leaching to the liquid phase during hydrogenolysis, where strong evidence on the SMSI that was hypothesized must be gathered in the following sections.

### Morphological Properties

#### Scanning electron microscopy

**Figure 1:**
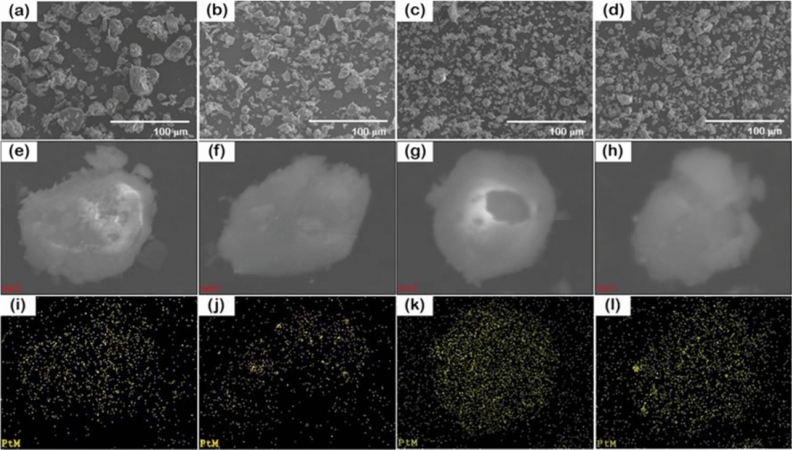
SEM images of (**a**,**e**) fresh Pt/γ-Al_2_O_3_, (**b**,**f**) reused Pt/γ-Al_2_O_3_, (**c**,**g**) fresh Pt/WO_x_/γ-Al_2_O_3_, (**d**,**h**) reused Pt/WO_x_/γ-Al_2_O_3_, the elemental distribution via SEM–EDX of (**i**) fresh Pt/γ-Al_2_O_3_, (**j**) reused Pt/γ-Al_2_O_3_, (**k**) fresh Pt/WO_x_/γ-Al_2_O_3_, (**l**) reused Pt/WO_x_/γ-Al_2_O_3_. Note that the SEM images (**e**) to (**h**) correspond to the elemental analysis in (**i**) to (**l**), and yellow dots represent the Pt element.

The morphology and surface elemental distribution of fresh and reused catalysts in both Pt/γ-Al_2_O_3_ and Pt/WO_x_/γ-Al_2_O_3_ systems are analyzed via SEM–EDX technique, as shown in Fig. 1. For the Pt/γ-Al_2_O_3_ catalyst, the EDX profile suggested a high dispersion of Pt on γ-Al_2_O_3_ in both fresh and reused catalyst shown in Fig. 1i,j, respectively. However, the EDX profile of Pt atom after the first reuse (Fig. 1j) exhibited some Pt clusters suggesting the agglomeration of Pt active site. Such agglomeration is the evidence of the catalyst deactivation found in the Pt/γ-Al_2_O_3_ in addition to a deactivation from Pt leaching confirmed via the ICP analysis in the previous section. On the other hand, for fresh and reused Pt/WO_x_/γ-Al_2_O_3_ catalysts, the EDX profiles depicted in Fig. 1k,l were similar, suggesting that doping with WO_x_ helped resist Pt agglomeration and also leaching. However, the resistance to agglomeration was not so clear via EDX profile. Thus, the role of WO_x_ on agglomeration was investigated via XRD in the following section.

#### X-ray diffraction analysis

**Figure 2:**
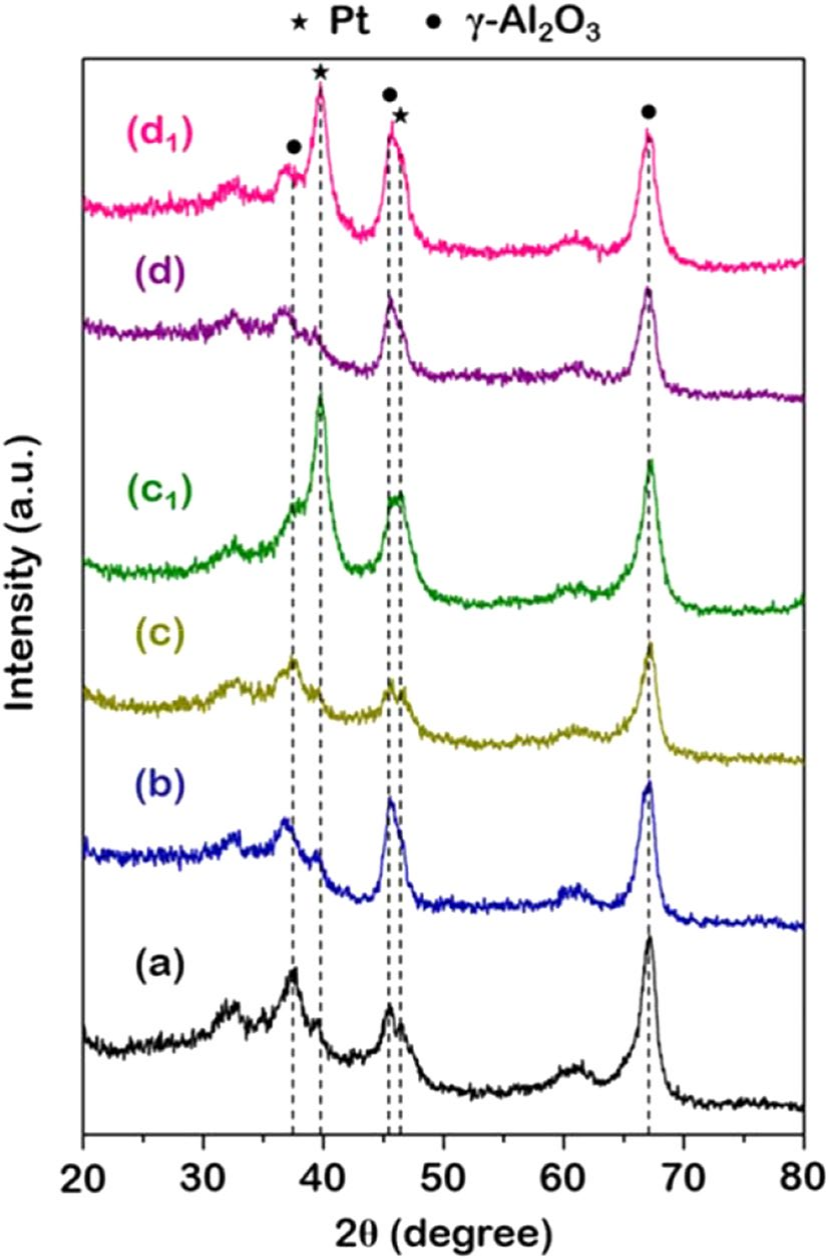
XRD patterns of (**a**) γ-Al_2_O_3_, (**b**) WO_x_/γ-Al_2_O_3_, (**c**) fresh Pt/γ-Al_2_O_3_, (**c**_**1**_) reused Pt/γ-Al_2_O_3_, (**d**) fresh Pt/WO_x_/γ-Al_2_O_3_, and (**d**_**1**_) reused Pt/WO_x_/γ-Al_2_O_3_.

Since the EDX profile suggested leaching resistance found in the system with WO_x_ but gave unclear information on agglomeration, the XRD profiles of the used catalysts were analyzed for both systems: Pt/γ-Al_2_O_3_ and Pt/WOx/γ-Al_2_O_3_ as in Fig. 2. The characteristic diffraction peaks of pristine γ-Al_2_O_3_ support have been reported at 2θ = 37.5°, 45.4° and 67.0°^38^, whereas the peaks for Pt metal have been reported at 2θ = 39.9° and 46.4°^30^. Moreover, the monoclinic WO_3_ (*m*-WO_3_) shows the unique diffraction peaks at 2θ = 23.6° and 33.5°^39^. First, the characteristic diffraction peaks of WO_x_ species over fresh WO_x_/γ-Al_2_O_3_ (Fig. 2b) were not observed in this study, which can be explained via the high dispersion of WO_x_ species on Al_2_O_3_ support^39,40^. For Pt/γ-Al_2_O_3_ and Pt/WO_x_/ γ-Al_2_O_3_ catalysts, the XRD diffraction peaks of Pt in fresh catalysts (Fig. 2c,d) were not detected, which is also due to the high dispersion of Pt metal on the γ-Al_2_O_3_ surface^40^ and WO_x_/γ-Al_2_O_3_.

The average particle size of adsorbed Pt can be determined via XRD using the Scherrer equation, which is:

1 $${\text{D}} = \frac{\text{K}\lambda}{{\beta}{\text{cos}}{\theta}}$$

where D is an average particle size in nm, λ is the X-ray wavelength, which is equal 0.154 nm, β is the peak width of the diffraction peak profile at half maximum height resulting from small crystallite size in radians unit, θ is peak position which is calculated form 2θ in radians unit and K is a Scherrer constant, normally taken as 0.9 for a good approximation.

The average particle sizes of Pt in Pt/γ-Al_2_O_3_ and Pt/WO_x_/γ-Al_2_O_3_ are 0.123 and 0.104 nm, respectively, which is close to the atomic radius of the Pt atom^41^. Therefore, constructions of Pt/γ-Al_2_O_3_ and Pt/WO_x_/γ-Al_2_O_3_ by using single Pt are selected in this DFT section.

On the reused catalysts, the Pt diffraction peak appeared at 2θ of 39.9° in both Pt/γ-Al_2_O_3_ and Pt/WO_x_/γ-Al_2_O_3_, as shown in Fig. 2c_1_,d_1_. Therefore, the presence of such a peak in the reused catalyst indicated the growth of Pt atoms into larger clusters. Hence, both the systems with and without the WO_x_ promoter, the agglomeration of Pt, is still present. Therefore, the Pt leaching played a critical role in the determination of the catalytic activity. Moreover, evidence from EDX and XRD could not give a clear picture of what parameter influenced the 1,3-PDO selectivity in the system with WO_x_. Thus, it was hypothesized that the changes in surface acidity when WO_x_ was added might contribute to the increase in 1,3-PDO production as it was reported that the Brønsted acid site correlated directly to the selectivity of such products^39^. As a result, the following section investigated the surface acidity in terms of Lewis and Brønsted acid sites to confirm the role of WO_x_ on desired product selectivity.

#### FTIR of adsorbed pyridine

**Figure 3:**
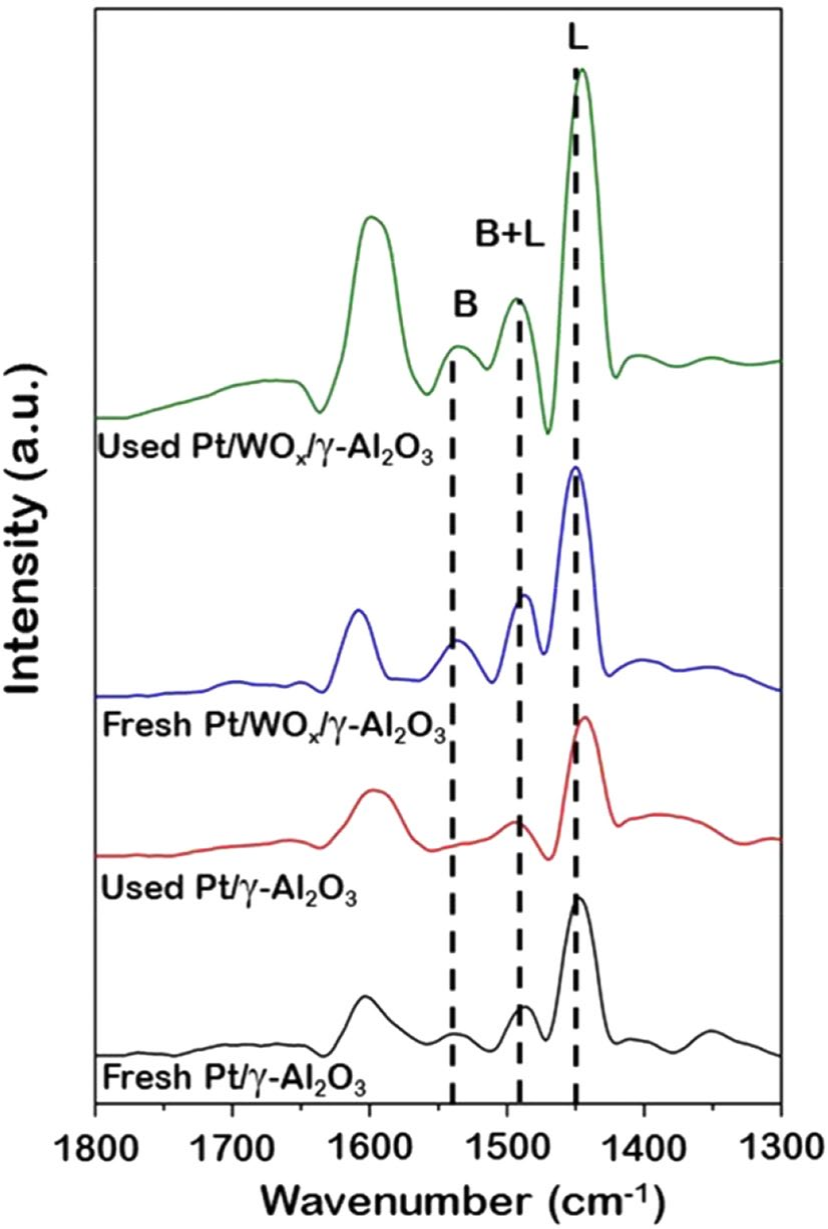
FTIR of pyridine adsorbed on fresh and reused Pt/γ-Al_2_O_3_ and Pt/WO_x_/γ-Al_2_O_3_ catalysts, where L and B represent Lewis and Brønsted acid.

To verify the hypothesis that WO_x_ is involved in an increased 1,3-PDO selectivity due to increasing Brønsted acidity, the Fourier-transform infrared spectroscopy (FTIR) combined with pyridine adsorption analysis was performed to determine the amount of Brønsted and Lewis acid sites on both Pt/γ-Al_2_O_3_ and Pt/WO_x_/γ-Al_2_O_3_ catalysts, as shown in Fig. 3. Peaks relating to the Lewis acid sites (denoted as L) were reported to be in the range between 1450 and 1614 cm^−1^^39,41^, whereas the peaks between 1540 and 1640 cm^−1^ corresponded to the Brønsted acid site (denoted as B)^42^. Moreover, the peak specifically at 1490 cm^−1^ represented the mixed Brønsted-Lewis acid sites (denoted as B + L) of the catalysts^43^. In this work, in all catalyst systems, the peak intensity of the Lewis acid site was much stronger than that of the Brønsted acid site, indicating that the Lewis acid site dominated the catalyst surface. It has been mentioned that the amount of Brønsted acidity determined the selectivity of the catalyst towards 1,3-PDO^30,39,44,45^. Our systems with a low amount of Brønsted acid site with low 1,3-PDO selectivity reflected through the parameter, as shown in Table 2, the ratio between Brønsted and Lewis acid site (B/L). In the case of fresh catalysts (black and blue lines), the lower 1,3-PDO selectivity on Pt/γ-Al_2_O_3_ than on Pt/WO_x_/γ-Al_2_O_3_ can be correlated to the lower amount of Brønsted acid site found in Pt/γ-Al_2_O_3_. Due to this observation, the selectivity has been promoted by the addition of WO_x_ to the Pt/γ-Al_2_O_3_ catalyst. Incorporation of WO_x_ not only increased the selectivity but also help preserve the Brønsted acid site on the surface as evident from the profile of the used catalyst (red and green lines), in which the reused Pt/WO_x_/γ-Al_2_O_3_ catalyst experienced a lower reduction in the amount of Brønsted acid site compared to the reused Pt/γ-Al_2_O_3_. Up to this point, it has been verified that the increase in 1,3-PDO selectivity was caused by an increase in the Brønsted acid site when the WO_x_ was present. Besides, the WO_x_ promoter improved the activity together with the improved stability via lower Pt leaching. However, the underlying phenomena that explain how WO_x_ increases catalyst performance in terms of activity, selectivity, and stability observed in the experimental results, are still needed to be investigated. Consequently, the computational technique based on DFT was used to provide insights into such phenomena in the following section.

**Table 2:** Catalytic performance and acidity of catalysts by FTIR.

Catalyst	Glycerol conversion (%)	Selectivity (%)		Acid sites (a.u./g_cat_)		B/L ratio^a^
		1,3-PDO	1,2-PDO	Brønsted	Lewis	
Pt/γ-Al_2_O_3_ (fresh)	23.2	6.9	18.7	0.23	1.41	0.16
Pt/γ-Al_2_O_3_ (used)	12.7	4.6	32.7	0.11	1.18	0.09
Pt/WO_x_/γ-Al_2_O_3_ (fresh)	35.8	29.0	10.2	0.52	2.02	0.26
Pt/WO_x_/γ-Al_2_O_3_ (used)	30.0	22.8	11.6	0.76	3.40	0.22

^a^Proportional concentration of Brønsted and Lewis acid sites.

### The activity of γ-Al_2_O_3_ and WO_x_/γ-Al_2_O_3_ surfaces via DOS

**Figure 4:**
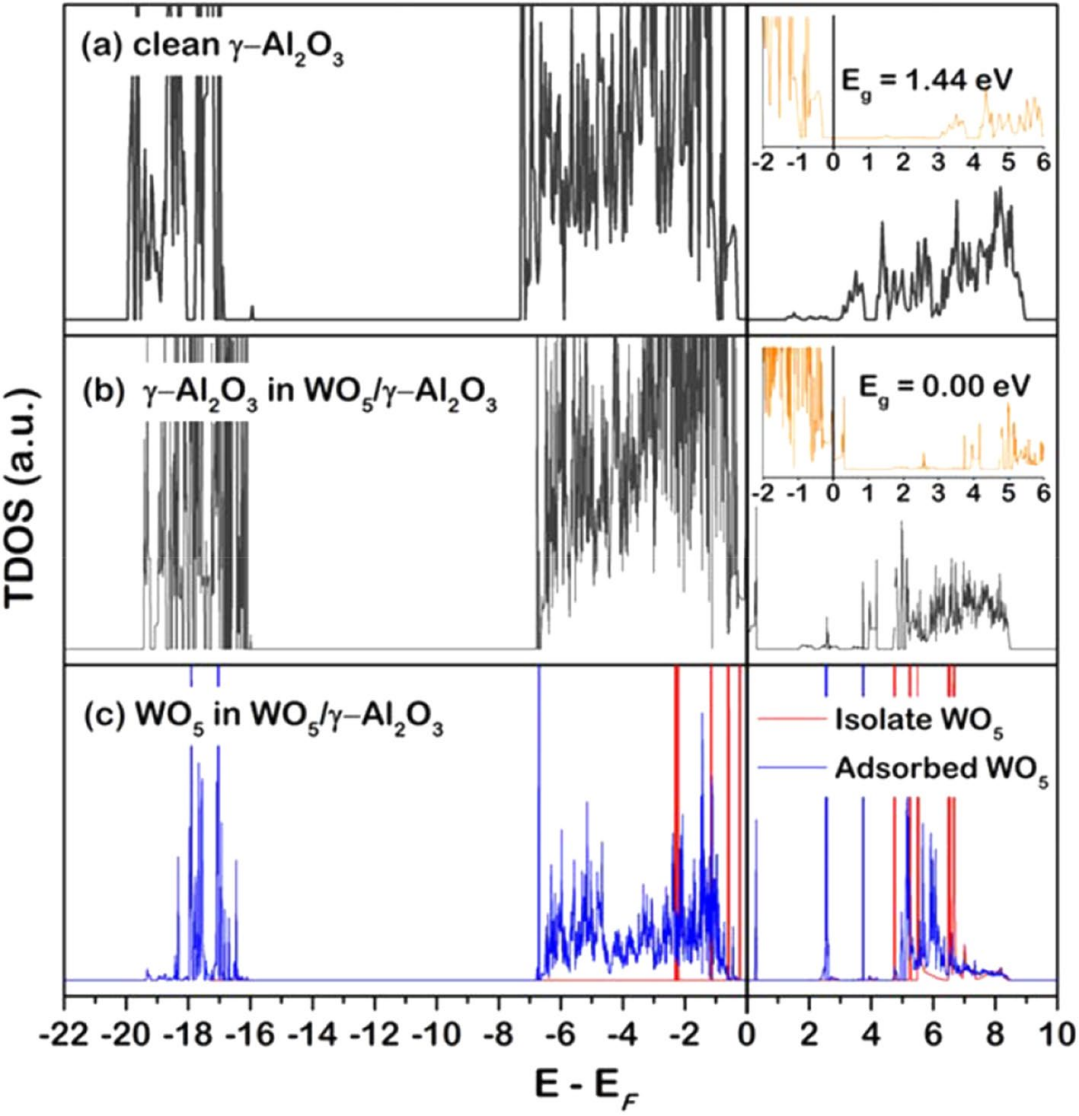
TDOS of the top layer (**a**) γ-Al_2_O_3_, (**b**) WO_5_/γ-Al_2_O_3,_ and (**c**) WO_5_ species before and after adsorption (red and blue, respectively), where the inlet graphs in (**a**) and (**b**) are plotted in a specific range from − 2.0 to 6.0 eV to show the energy gap clearly.

As various evidence has revealed the role of WO_x_ on the increased catalytic performance in terms of activity, selectivity, and stability, this section provides insights via DFT-based analyses on how the WO_x_ modified the properties of the catalyst. The effects of WO_x_ on the catalyst surface were investigated, comparing the changes in the electronic property of γ-Al_2_O_3_ and WO_5_-modified γ-Al_2_O_3_ models via the density of state (DOS) analysis. The total DOS (TDOS) profile of the top layer of γ-Al_2_O_3_ and WO_5_/γ-Al_2_O_3_ surfaces, as well as the isolated and adsorbed WO5 on γ-Al2O3 surface, are plotted as shown in Fig. 4. The use of WO_5_ to represent WO_x_ species was discussed in the slab information in computational details Sect. 2.4.2. All TDOS profiles are plotted in the range between − 22.00 to 10.00 eV, where the negative and positive zones represent bonding and antibonding divided at the Fermi energy (E_F_) at 0.00 eV. The energy gap (E_g_) of 1.44 eV of γ-Al_2_O_3_ surface being calculated only from the top layer to confirm the model reliability was in good agreement with Dinge et al.^46^. In the WO_x_ modified surface, the TDOS of γ-Al_2_O_3_ upshifted towards the antibonding zone shown in Fig. 4b. The improved activity, when the promoter is present, was caused by additional electronic states or interstates between the valence band maximum (VBM) and the conduction band minimum (CBM), resulting in reduced energy gap. Thus, this suggested a higher tendency of the γ-Al_2_O_3_ towards Pt adsorption.

Moreover, the majority of the TDOS profile of adsorbed WO_5_ species on γ-Al_2_O_3_ surface in Fig. 4c exhibited to overlap with the TDOS peaks of γ-Al_2_O_3_ surface, signifying strong interaction of such species with the support. Hence, it was observed that the WO_x_ species modified the electronic property of the support leading to an increase in activity in terms of the bonding of the adsorbates to the surface. In addition to the effect of such a species on the support, its effects on the Pt active site is still to be explained. Thus, the next section investigated the effects of the WO_x_ on the Pt active site and how it modified the activity and stability of Pt.

### Metal–support-promoter interactions

**Figure 5:**
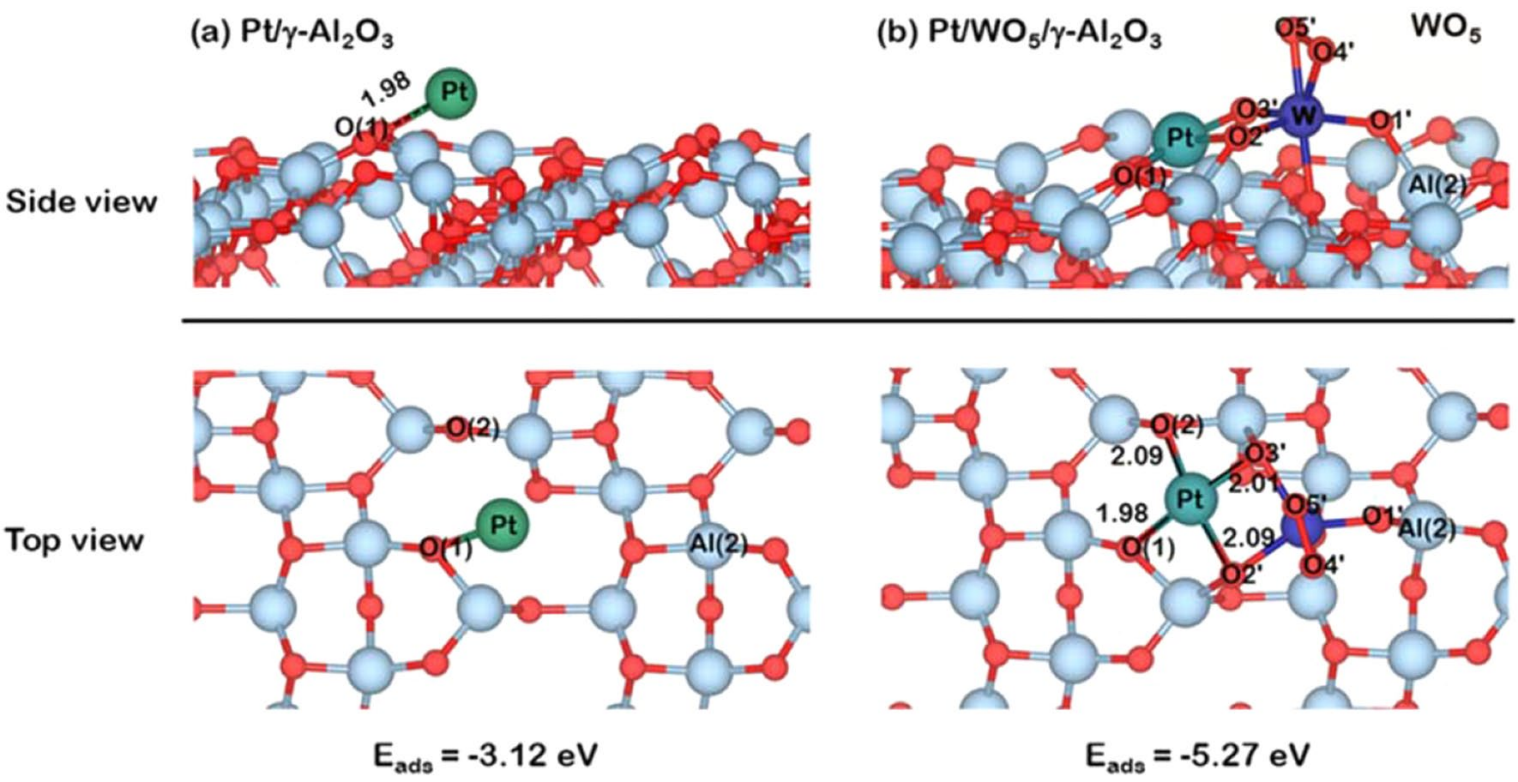
Top and side views of the most stable adsorption structures of Pt atom on (**a**) γ-Al_2_O_3_ and (**b**) WO_5_/γ-Al_2_O_3_ surfaces. Note that the atomic radius of each atom is modeled to its isolated atomic radius.

Based on the hypothesis that the strong metal–support interaction (SMSI) would reduce the leaching of the active metal, promoting the stability of the catalyst, this part investigated the role of WO_x_ species on the stability of Pt active site supported on γ-Al_2_O_3_ from the theoretical point of view based on DFT. The metal-support interaction (MSI) between Pt and γ-Al_2_O_3_ as well as WO_5_/γ-Al_2_O_3_ surfaces can be described by considering adsorption energies of Pt on γ-Al_2_O_3_ and WO_5_/γ-Al_2_O_3_ surfaces. The most stable adsorption configuration of Pt atom, significant bond distances of Pt on γ-Al_2_O_3_ and WO_5_/γ-Al_2_O_3_ surfaces, and adsorption energy (E_ads_) are reported in Fig. 5. For the adsorption of Pt on γ-Al_2_O_3_ surface, a Pt atom preferentially adsorbed on the hollow site of γ-Al_2_O_3_ surface in zone IV depicted in Figs. 7c and 5a with an adsorption strength (E_ads_) of ‒ 3.12 eV, in which the distance between Pt and O(1) is 1.98 Å. For adsorption of Pt on WO_5_/γ-Al_2_O_3_ surface shown in Fig. 5b, the Pt atom also favored the hollow site at zone IV with the E_ads_ of ‒ 5.27 eV, which is 70% stronger than that on γ-Al_2_O_3_. The strong interaction between Pt and WO_5_/γ-Al_2_O_3_ surface can be described via the formation of four bonds; two bonds between Pt and O of γ-Al_2_O_3_, which are Pt-O(1) and Pt-O(2) with the bond length of 1.98 Å and 2.09 Å, respectively and the other two bonds between Pt with O2′ and O3′ of the WO_5_ species with bond distances of 2.09 Å for Pt-O2′ and 2.01 Å for Pt and O3′. These results indicated that with the presence of WO_5_ on the γ-Al_2_O_3_ surface, the Pt atom could be adsorbed strongly on the support since more bonds were formed, promoting SMSI. Therefore, a low Pt leaching found in the Pt/WO_x_/γ-Al_2_O_3_ is due to SMSI promoted through the WO_x_ species that increases more bonding with Pt in addition to the bonding between Pt and Al_2_O_3_ support.

**Figure 6:**
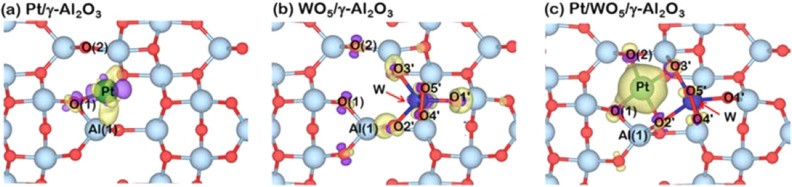
Charge density differences of (**a**) Pt atom adsorption on γ-Al_2_O_3_ surface, (**b**) WO_5_ molecule adsorption on γ-Al_2_O_3_ surface, and (**c**) Pt atom adsorption on WO_5_/γ-Al_2_O_3_ surface presented with isovalue of ± 0.015 e Å^−3^. The electron accumulation and depletion are represented by yellow and violet regions, respectively. Note that the atomic radius of each atom is modeled to its isolated atomic radius.

Having observed that the WO_x_ strengthens the Pt-support bonding promoting SMSI through the Pt binding energy to the support, this section probed the changes in surface electronic property, especially at the Pt active site via the charge density difference together with Bader charge analysis. For the notation of the charge density difference profile illustrated in Fig. 6, the charge accumulation and depletion region are labeled in yellow and violet, respectively. The Bader charge changes of each component summarized in Table 3 defined negative and positive values of Bader charge change as electrons gain and loss, respectively. To clarify via charge analysis on each interaction between metal, support, and the WO_x_ species, three models in Fig. 6a–c were analyzed, where the model (a) would reveal the metal-support interaction without WO_x_ promoter, while model (b) gave the information about the WO_x_–support interaction, and model (c) reflected the role of WO_x_ in the 3-way interaction of metal–support-WO_x_. The first information about the metal-support, Pt and Al_2_O_3_, suggested that the direction of electron transfer is from Pt to Al_2_O_3_ support verified by the electron gain of -0.28|e| in the support and electron loss of + 0.28|e| from Pt as shown in Table 3. Besides, the charge density difference for this system revealed that the metal-support bonding occurred only between Pt and the O(1) site. On the WO_x_–support interaction, the electron moved in the opposite direction—from the support Al_2_O_3_ to the WO_x_ species as the electron gain to the species up to -2.33|e| and a loss of + 2.33|e| from the support. Interestingly, the WO_x_ species acting as an electron acceptor withdrew electrons from two nearby oxygen atom sites at O(2) and another oxygen connecting to the Al(1) site. The electron deficit of more oxygen sites on the surface would lead to a higher tendency in the acceptance of the incoming electron-rich species; thus, the Pt atom if adsorbed onto such a WO_x_-modified surface, may exhibit strong metal-support interaction. The Pt/WO_x_/γ-Al_2_O_3_ model in Fig. 6c determined the WO_x_ species to be even a stronger electron acceptor than when the Pt is absent. The WO_x_ species now gain up to − 2.91|e|, while the electron loss in support is lower than that in the case without Pt with the value of + 1.77|e| and the Pt atom transfers the electron to the WO_x_ species losing + 1.14|e| which is much higher than when it was in the Pt/γ-Al_2_O_3_ system. Considering the charge density changes in this WO_x_-modified system, the Pt atom adsorbed with a high number of the contact point to both the support and WO_x_ species compared to a single contact point of Pt to only Al_2_O_3_ surface found in the Pt/γ-Al_2_O_3_ system, suggesting the existence of the strong metal-support-promoter interaction or the SMSPI. Therefore, the SMSPI found in the Pt/WO_5_/γ-Al_2_O_3_ surface can be described in terms of electron transfer that the WO_x_ species acting as a strong electron acceptor led to an increase in contact points of Pt and the support. Hence, the SMSPI is the key in the resistance towards lower Pt leaching during the hydrogenolysis process resulting in a catalyst with high stability and should be the root cause of high activity in terms of glycerol conversion and high 1,3-PDO selectivity.

**Table 3:** Bader charge change of Pt, WO_5_ and the γ-Al_2_O_3_ component in the Pt/γ-Al_2_O_3_, WO_5_/γ-Al_2_O_3_, and Pt/WO_5_/γ-Al_2_O_3_ surfaces.

System	Analyzed component	Bader charge change |e|
Pt/γ-Al_2_O_3_	Pt	+ 0.28
	γ-Al_2_O_3_	‒ 0.28
WO_5_/γ-Al_2_O_3_	WO_5_	‒ 2.33
	γ-Al_2_O_3_	+ 2.33
Pt/WO_5_/γ-Al_2_O_3_	Pt	+ 1.14
	WO_5_	‒ 2.91
	γ-Al_2_O_3_	+ 1.77

## Conclusions

The investigation on the roles of WO_x_ on increased activity, selectivity, and stability in the Pt/WO_x_/γ-Al_2_O_3_ catalyst during the glycerol hydrogenolysis reaction can be summarized as follows. On the enhanced catalytic activity, introducing WO_x_ led to a 35% increase in glycerol conversion, comparing the fresh Pt/WO_x_/γ-Al_2_O_3_ and Pt/γ-Al_2_O_3_ catalyst. The improved activity can be explained via the total density of states (TDOS) profile of the WO_x_-modified Pt/γ-Al_2_O_3_ surface, suggesting that the WO_x_ promoter lowered the energy gap (E_g_) by upshifting the TDOS of the system towards the antibonding region and forming the additional electronic states or interstates between the VBM and CBM. Moreover, a high catalyst selectivity towards 1,3-PDO found in the Pt/WO_x_/γ-Al_2_O_3_ catalyst was due to the increase in the number of Brønsted acid sites confirmed via FTIR, which directly linked to the selective conversion of the glycerol to 1,3-PDO. In the WO_x_-modified Pt/γ-Al_2_O_3_ catalyst, evidence from the first-principle point-of-view revealed the interaction between the added WO_x_ and the Pt active site was strengthened to result in stronger Pt adsorption to the support. This resulted in the strong metal-support-promoter (WO_x_) interaction (SMSPI) and stability of Pt against leaching. Charge density difference and Bader charge analysis suggested that more electrons transferred from both the Al_2_O_3_ support and Pt active site to WO_x_ than those between Al_2_O_3_ and Pt in the Pt/γ-Al_2_O_3_ system. As for the proposed design guidelines for an active-selective-stable Pt/γ-Al_2_O_3_ catalyst, the improved activity when the promoter is present is caused by the interstates reducing the energy gap. Thus, the promoter that promotes the interstates should improve activity. On the selectivity, any promoter that promoted Brønsted acid site formation would enhance 1,3-PDO selectivity. For the stability, especially in terms of the resistance against Pt leaching, the evidence suggested the SMSI be the main impact towards such deactivation. Hence, the promoter that promoted SMSI or, preferably, the SMSPI would lead to a highly stable catalyst for the hydrogenolysis of glycerol.

## Methodology

### Catalyst preparation

#### Preparation of Pt/γ-Al_2_O_3_ catalyst

The Pt/γ-Al_2_O_3_ catalyst was prepared via wet impregnation. First, laboratory-grade γ-Al_2_O_3_ powder (from KemAus) was calcined at 900 °C for 3 h. The γ-Al_2_O_3_ support of 2.0 g was impregnated using 0.105 g chloroplatinic acid hydrate (H_2_PtCl_6_·nH_2_O) as the platinum precursor forming 5 wt.% Pt/γ-Al_2_O_3_. The γ-Al_2_O_3_ and H_2_Cl_6_Pt·nH_2_O were mixed in deionized (DI) water with the ratio of 15 mL DI water per 1 g of Pt precursor, and the mixture was stirred at 500 rpm for 16 h under an ambient condition until homogenously mixed before drying at 110 °C overnight. Finally, the dried sample designated as Pt/γ-Al_2_O_3_ was calcined under airflow at 300 °C for 3 h.

#### Preparation of WO_x_/γ-Al_2_O_3_ support

For the WO_x_-modified γ-Al_2_O_3_ support, the wet impregnation method was performed. First, the ammonium metatungstate hydrate (AMT) weighed 0.446 g was dissolved in 25 mL DI water before being stirred at room temperature (30 °C) until homogeneously mixed. After that, the calcined γ-Al_2_O_3_ powder of 3.0 g was introduced into the AMT solution, and the mixture was continuously stirred at 500 rpm for 16 h to impregnate the WO_x_ promoter. Finally, the solution was dried at 110 °C overnight before being calcined under airflow at 900 °C for 3 h resulting in a WO_x_/γ-Al_2_O_3_ denoted as 10WO_x_/Al_2_O_3_.

#### Preparation of Pt/WO_x_/γ-Al_2_O_3_ catalyst

Introduction of Pt onto the WO_x_/γ-Al_2_O_3_ support to form 5 wt%Pt/WO_x_/γ-Al_2_O_3_ catalyst was also prepared via the wet impregnation method. Similarly, 0.105 g of the chloroplatinic acid hydrate was used as Pt precursor and homogeneously dissolved in DI water with the ratio of 15 mL DI water/g-Pt-precursor and stirred at room temperature (30 °C) at 500 rpm. Afterward, 2.0 g of calcined WO_x_/γ-Al_2_O_3_ powder was added to the prepared chloroplatinic acid solution. The mixture was stirred at 500 rpm under the ambient condition (30 °C, 1 atm) for 16 h. After that, the Pt/WO_x_/γ-Al_2_O_3_ catalyst precursor was dried at 110 °C overnight to eliminate the solvent. Subsequently, the dried Pt/WO_x_/γ-Al_2_O_3_ powder was calcined at 300 °C under airflow for 3 h. The loading of Pt and WO_x_ were 5 wt% and 10 wt%, respectively.

### Catalyst characterizations

#### Chemical analysis

The inductively coupled plasma optical emission spectroscopy (ICP-OES) technique performed on a 2100 DV from Perkin Elmer was used to measure the amount of Pt and W elements leaching from the surface of Pt/γ-Al_2_O_3_ and Pt/WO_x_/γ-Al_2_O_3_ catalysts to the liquid phase during hydrogenolysis reaction. The 1 mL of sample solution withdrawn from the reaction solution after the hydrogenolysis reaction was diluted in 4 mL of DI water, where the energy profile was used for the measurement.

#### Morphological analysis

Morphological properties, including crystallinity, structure, and behavior of Pt on γ-Al_2_O_3_ and WO_x_/γ-Al_2_O_3_ support, were determined using (1) the Powder X-ray diffraction (XRD) measurement via Bruker D8 Advance using Cu Kα irradiation in the 2θ range of 20° to 80° with a step size of 0.05° s^-1^, and (2) the Scanning Electron Microscope (SEM) equipped with a silicon-drift detector to collect the energy-dispersive X-ray (EDX) performed via the Hitachi model S-3400 N for SEM, and EDAX model Apollo X for SEM–EDX.

#### Surface properties of Pt on γ-Al_2_O_3_ and WOx/γ-Al_2_O_3_

The different types of acid sites, Brønsted and Lewis on the catalyst surface, were determined using Fourier-transform infrared spectroscopy (FTIR) of adsorbed pyridine via a Bruker Equinox 55 FT-IR spectrometer equipped with a mercury cadmium telluride detector. The sample of 0.055 g was dehydrated under vacuum at the isothermal condition of 300 °C for 1 h before being settled down to 50 °C. Next, a known amount of gaseous pyridine was introduced into the chamber at a temperature of 50 °C and held for 20 min, where the unadsorbed pyridine was removed by the vacuum pump. The FTIR spectra were recorded by the adsorbed pyridine on sample surfaces.

### Catalytic reaction testing

The catalytic properties of a catalyst during glycerol hydrogenolysis were investigated with a catalyst weighed 0.60 g (5%Pt/γ-Al_2_O_3_ and 5%Pt/WO_x_/γ-Al_2_O_3_) together with 0.36 g of glycerol in 11.64 g DI water. The mixture was introduced to a 100-mL stainless steel autoclave, which was used as a reactor. Then, the flushing of the reactor with H_2_ was performed three times to remove air before being pressurized to 0.5 MPa with H_2_. The mixtures were subsequently heated to 140 °C with a constant stirring speed of 800 rpm. The reaction was continuously operated for 6 h. After the reaction, the reactor was immediately cooled down to an ambient temperature of 30 °C and held for 12 h to make sure that all components in gas and liquid phases were condensed and in an equilibrium state. Next, the pressure was released, and the 0.30 g of ethylene glycol was added as an internal standard for gas chromatography analysis. The crude product was removed and sent to the separation process, where the slurry crude product was centrifuged to separate solid crude from the liquid. The solid crude was washed with DI water ten times and dried at 110 °C for 12 h until no weight change can be detected to test the reusability of the catalyst. The recovered catalyst was introduced as the second batch used catalyst (2^nd^ reused) only after the recovery process was performed.

For the separated liquid, it was analyzed for hydrogenolysis products using Shimadzu 14B gas chromatography equipped with DB-WAX-UI capillary column (30 m × 0.32 mm × 0.5 μm) with a flame ionization detector (GC-FID). The conversion of glycerol and selectivity for each possible liquid product, including 1,2-PDO, 1-propanol (1-PrOH), 2-propanol (2-PrOH), and 1,3-PDO, were calculated using the following equations, respectively.

2 $$\text{Conversion }({\%})=\frac{\text{moles of glycerol }\left({\text{in}}\right) \, \text{- moles of glycerol (out)}}{\text{moles of glycerol (in)}}\times100$$

3 $$\text{Selectivity }\left({\%}\right)=\frac{\text{moles of product}}{ \text{moles of glycerol }\left({\text{in}}\right) \, \text{- moles of glycerol (out)}}\times100$$

### Computational details

#### DFT calculations

The spin-polarized DFT calculations^47^ were performed via the Vienna ab initio simulation package version 5.4.4 (VASP)^48,49^, where the projector augmented wave (PAW) potential^50,51^ was employed to represent the interaction between valence electrons and the ion core, while the exchange–correlation functional along with the generalized gradient approximation (GGA) by Perdew, Burke, and Ernzerhof (PBE) was used^52^. The 400 eV of cut-off energy with the convergence criteria of 1.0 × 10^–6^ eV/atom were set for the minimization. The 5 × 3 × 1 of the Monkhorst–Pack grid was used to construct the Brillouin-zone^53^. The geometry optimization was performed within the conjugate gradient method^54^. All structures were relaxed until the force convergence is lower than 0.05 eV/Å. The partial occupancies were determined via the Gaussian smearing^55^ of 0.05 eV smearing width. Moreover, the Van der Waals dispersion correction term of DFT-D3 method proposed by Grimme et al*.*^56^ was also adopted.

The interactions between a platinum (Pt) atom on different surfaces of γ-Al_2_O_3_ and WO_5_/γ-Al_2_O_3_ supports were determined through an adsorption energy (E_ads_) calculation as follows.

4 $${\text{E}}_{{{\text{ads}}}} = {\text{ E}}_{{\text{Pt-surface}}}- {{ (E}}_{{{\text{Pt}}}} + {\text{E}}_{{{\text{surface}}}} {)}$$

The ${\text{E}}_{\text{Pt-surface}}$ parameter refers to the total energy of the adsorption complex (Pt atom on γ-Al_2_O_3_ and WO_x_/γ-Al_2_O_3_ surfaces), ${\text{E}}_{\text{Pt}}$ denotes the total energy of an isolated Pt atom in a vacuum, and E_surface_ is the total energy of the clean γ-Al_2_O_3_ and WO_x_/γ-Al_2_O_3_ surfaces. A negative value of ${\text{E}}_{\text{ads}}$ indicates that the adsorption process is possible and suggests an attractive interaction between Pt and γ-Al_2_O_3_ as well as WO_x_/γ-Al_2_O_3_ surfaces. Charge distribution on adsorbed Pt and γ-Al_2_O_3_ support, as well as WO_x_/γ-Al_2_O_3_, can be described using partial charge analysis ${(\Delta}{\delta}_{\text{Pt,Sur}}$), whose partial charge is calculated based on Bader charge analysis^57–60^ as follows:

5 $${\Delta}{\delta}_{\text{Pt,Sur}} = {\delta}_{\text{P,Sur}}^{\text{ads}}- {\delta}_{\text{Pt,Sur}}^{\text{clean}}$$

The ${\delta}_{\text{Pt,Sur}}^{\text{clean}}$ denotes the partial charge of isolated Pt atom and support before Pt adsorption while the ${\delta}_{\text{Pt,Sur}}^{\text{ads}}$ represents the partial charge of adsorbed Pt and support after the adsorption process.

#### Slab information

**Figure 7:**
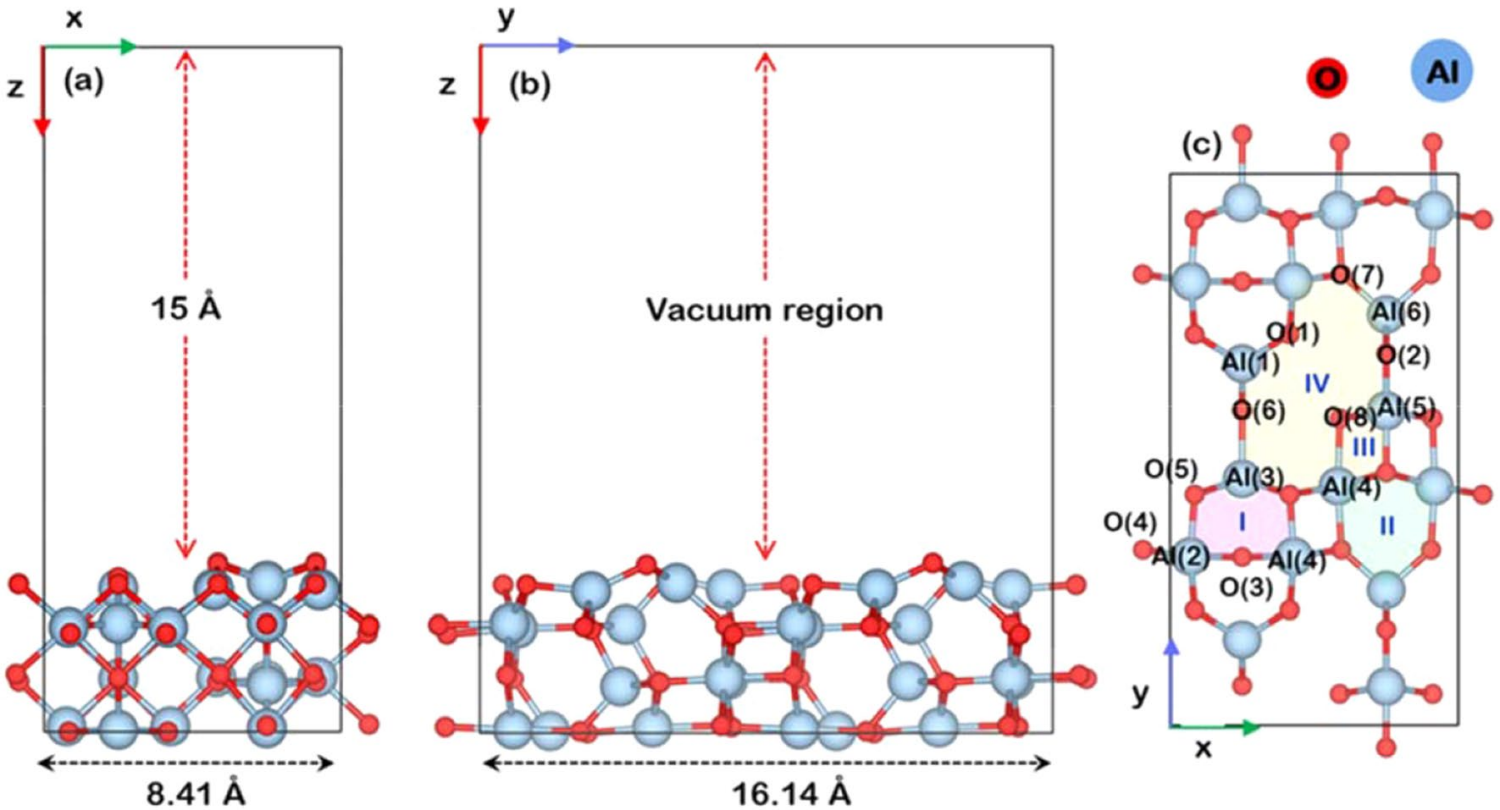
Surface model of γ-Al_2_O_3_(110) projected (**a**) along the (100)-direction, (**b**) along the (010)-direction, and (**c**) along (001)-direction. The vacuum of the slab is set to 15 Å. The zones of the possible active site in (**c**) are classified into the zone I, II and IV as labeled in magenta, green, and yellow, respectively. Note that the atomic radius of each atom is modeled to its isolated atomic radius.

The model information of clean γ-Al_2_O_3_ surface employed in this study is based on the work from Dinge et al*.*^46,61^*,* as shown in Fig. 7a,b. Four atomic layers of γ-Al_2_O_3_ (110) with a dimension of 8.41 Å × 16.14 Å × 19.41 Å were cleaved from the optimized bulk structure of γ-Al_2_O_3_ consisting of 32 Al atoms and 48 O atoms. A vacuum along the z-axis of 15 Å was added to avoid interactions between the slab caused by its periodicity. During calculations, the two bottom layers were fixed to their lattice position, while the upper two layers and adsorbed species were allowed to fully relax. All possible adsorption sites are illustrated in Fig. 7c. The stable adsorption sites on γ-Al_2_O_3_ (110) surface have been previously investigated by Wang et al.^62^ but for Cu and Pd adsorptions. For the model of the WO_x_/γ-Al_2_O_3_ surface, the polytungstate form of WO_x_ (denoted as WO_5_ and WO_6_) has been reported as a highly effective form for producing 1,3 propanediol via hydrogenolysis of glycerol^29,30,63^. Therefore, the smallest structure size of the polytungstate species of WO_5_ was selected to represent the WO_x_ model on the γ-Al_2_O_3_ (110) surface for this computational investigation.

## Supplementary Information

Supplementary Information.
