# Supplementary material for: Experimental and computational study on roles of WOx promoting strong metal support promoter interaction in Pt catalysts during glycerol hydrogenolysis
Source: Sci Rep. 2021 Jan 12;11:530. doi: 10.1038/s41598-020-79764-3 (PMC7804099; doi:10.1038/s41598-020-79764-3)
Supplement: Supplementary file 1 — Supplementary Information. [file 41598_2020_79764_MOESM1_ESM.pdf]

Supplementary Document

Experimental and computational study on roles of WO_x promoting strong metal support promoter interaction in Pt catalysts during glycerol hydrogenolysis

Tinnakorn Saelee[1, 2,5‡], Poonnapa Limsoonthakul[2, 5‡], Phakaorn Aphichoksiri[1, 2, 5], Meena Rittiruam[1,2,6], Mongkol Lerdpongsiripaisarn[1, 2, 5], Takanori Miyake[3], Hiromi Yamashita[4], Kohsuke Mori[4], Yasutaka Kuwahara[4], Supareak Praserthdam[1, 2 *], Piyasan Praserthdam[2]

[1]High-Performance Computing Unit (CECC-HCU), Centre of Excellence on Catalysis and Catalytic Reaction Engineering (CECC), Department of Chemical Engineering, Faculty of Engineering, Chulalongkorn University, Bangkok 10330, Thailand
[2]Centre of Excellence on Catalysis and Catalytic Reaction Engineering (CECC), Department of Chemical Engineering, Faculty of Engineering, Chulalongkorn University, Bangkok 10330, Thailand
[3]Faculty of Environmental and Urban Engineering Department of Chemical, Energy and Environmental Engineering, Kansai University, Suita, Osaka 564-8680 Japan
[4]Division of Materials and Manufacturing Science, Graduate School of Engineering, Osaka University, 1-1 Yamadaoka, Suita, Osaka 565-0871 Japan
[5]Saelee Research Group, Chulalongkorn University, Bangkok 10330, Thailand
[6]Rittiruam Research Group, Chulalongkorn University, Bangkok 10330, Thailand

[‡]These authors contributed equally to this work
*Corresponding author e-mail: supareak.p@chula.ac.th (Dr. Supareak Praserthdam)

Contents **Page**

Table S1 The CONTCAR file of optimized geometry of clean γ-Al_2O_3 surface 2
Table S2 The CONTCAR file of optimized WO_5/ γ-Al_2O_3 surface 5
Table S3 The CONTCAR file of the most stable Pt on γ-Al_2O_3 surface 8
Table S4 The CONTCAR file of the most stable Pt on WO_5/γ-Al_2O_3 surface 11
Figure S1 The XPS of Pt4f electrons from the Pt/γ-Al_2O_3 and Pt/WOx/γ-Al_2O_3 14

Table S1 The CONTCAR file of optimized geometry of clean γ-Al_2O_3 surface

Al O

32 48

Selective dynamics

Direct

0.0702196164332059	0.3063030317546124	0.2094800044313158	T	T	T
0.5825432989590734	0.4342082833249682	0.1526924070342361	T	T	T
0.5749999720000005	0.1988699999999994	0.0080600000000004	F	F	F
0.0790000009999972	0.0729800009999977	0.0802899979999978	F	F	F
0.9250000279999995	0.1988699999999994	0.0080600000000004	F	F	F
0.4209999919999987	0.0729800009999977	0.0802899979999978	F	F	F
0.4297803905667982	0.3063030317546124	0.2094800044313158	T	T	T
0.9174567010409266	0.4342082833249682	0.1526924070342361	T	T	T
0.7500000000000000	0.0706619389282064	0.2090204994839193	T	T	T
0.7500000000000000	0.0665100009999975	0.0776999939999996	F	F	F
0.7500000000000000	0.3774399930000030	0.0028799999999976	F	F	F
0.2500000000000000	0.4419099979999999	0.0051800000000028	F	F	F
0.2500000000000000	0.4474344068688796	0.1588628313857612	T	T	T
0.2500000000000000	0.1558836012914766	0.2209594921672479	T	T	T
0.2500000000000000	0.2530299960000022	0.0756800030000022	F	F	F
0.7500000000000000	0.2453331903556145	0.1581244980545427	T	T	T
0.0726667075079656	0.8054882567802585	0.2060721478486899	T	T	T
0.5828080210088769	0.9340484675631520	0.1527911655091163	T	T	T
0.5749999720000005	0.6988699999999994	0.0080600000000004	F	F	F
0.0790000009999972	0.5729799639999982	0.0802899979999978	F	F	F
0.9250000279999995	0.6988699999999994	0.0080600000000004	F	F	F
0.4209999919999987	0.5729799639999982	0.0802899979999978	F	F	F
0.4273332994920387	0.8054882567802585	0.2060721478486899	T	T	T
0.9171919789911231	0.9340484675631520	0.1527911655091163	T	T	T
0.7500000000000000	0.5707587086076191	0.2090065034170535	T	T	T
0.7500000000000000	0.5665100379999970	0.0776999939999996	F	F	F
0.7500000000000000	0.8774399639999970	0.0028799999999976	F	F	F
0.2500000000000000	0.9419100269999987	0.0051800000000028	F	F	F
0.2500000000000000	0.9481999870000024	0.1594399929999994	F	F	F
0.2500000000000000	0.6559781332437079	0.2180934242850449	T	T	T
0.2500000000000000	0.7548542571129202	0.0679860130839496	T	T	T
0.7500000000000000	0.7453946594568156	0.1581512038980916	T	T	T

0.4059999979999986	0.4534200180000028	0.0846099999999979	F	F	F
0.9170000120000026	0.3119700080000030	0.0000000000000000	F	F	F
0.4150294667955253	0.4189355327422531	0.2160146654302074	T	T	T
0.9056347552465257	0.3183329180173845	0.1561024980615217	T	T	T
0.9112091060554397	0.0571388181303445	0.1441912362440053	T	T	T
0.4150346964325772	0.2092977076389054	0.2472489023825027	T	T	T
0.9059999979999986	0.0783400010000008	0.0109400010000016	F	F	F
0.4160000030000006	0.1855500019999994	0.0739599959999993	F	F	F
0.5887908939445603	0.0571388181303445	0.1441912362440053	T	T	T
0.0849653175674240	0.2092977076389054	0.2472489023825027	T	T	T
0.5940000020000014	0.0783400010000008	0.0109400010000016	F	F	F
0.0839999969999994	0.1855500019999994	0.0739599959999993	F	F	F
0.0939999949999972	0.4534200180000028	0.0846099999999979	F	F	F
0.5829999879999974	0.3119700080000030	0.0000000000000000	F	F	F
0.0849705332044747	0.4189355327422531	0.2160146654302074	T	T	T
0.5943652447534743	0.3183329180173845	0.1561024980615217	T	T	T
0.7500000000000000	0.4409900120000003	0.0794299969999983	F	F	F
0.7500000000000000	0.4592289560254974	0.2154063956520737	T	T	T
0.2500000000000000	0.0721436726139301	0.1578026131825576	T	T	T
0.2500000000000000	0.0603599989999992	0.0089200000000034	F	F	F
0.2500000000000000	0.3249400019999982	0.0025899999999979	F	F	F
0.2500000000000000	0.3052101768369376	0.1552991431137843	T	T	T
0.7500000000000000	0.1742308156883203	0.2251748456073362	T	T	T
0.7500000000000000	0.1905300040000029	0.0756800030000022	F	F	F
0.4059999979999986	0.9534199590000014	0.0846099999999979	F	F	F
0.9170000120000026	0.8119700080000030	0.0000000000000000	F	F	F
0.4146442352844595	0.9181289753737126	0.2155069116936531	T	T	T
0.9049276683336706	0.8186107523643894	0.1549573450770602	T	T	T
0.9111288824368533	0.5571802334525103	0.1441373009639900	T	T	T
0.4149040369562891	0.7090415073991260	0.2445606132495302	T	T	T
0.9059999979999986	0.5783400090000015	0.0109400010000016	F	F	F
0.4160000030000006	0.6855500019999994	0.0739599959999993	F	F	F
0.5888711175631467	0.5571802334525103	0.1441373009639900	T	T	T
0.0850959770437120	0.7090415073991260	0.2445606132495302	T	T	T
0.5940000020000014	0.5783400090000015	0.0109400010000016	F	F	F
0.0839999969999994	0.6855500019999994	0.0739599959999993	F	F	F
0.0939999949999972	0.9534199590000014	0.0846099999999979	F	F	F

0.5829999879999974	0.8119700080000030	0.0000000000000000	F	F	F
0.0853557647155404	0.9181289753737126	0.2155069116936531	T	T	T
0.5950723316663294	0.8186107523643894	0.1549573450770602	T	T	T
0.7500000000000000	0.9409899819999978	0.0794299969999983	F	F	F
0.7500000000000000	0.9592449822824574	0.2154811663926363	T	T	T
0.2500000000000000	0.5713291516563356	0.1566432698818830	T	T	T
0.2500000000000000	0.5603600319999984	0.0089200000000034	F	F	F
0.2500000000000000	0.8249400019999982	0.0025899999999979	F	F	F
0.2500000000000000	0.8052706592057087	0.1506610497785044	T	T	T
0.7500000000000000	0.6742838898626090	0.2251604210495892	T	T	T
0.7500000000000000	0.6905300190000006	0.0756800030000022	F	F	F

Table S2 The CONTCAR file of optimized WO_5/ γ-Al_2O_3 surface

Al O W

32 53 1

Selective dynamics

Direct

0.0764341604114130	0.3064099778335573	0.2073464916464912	T	T	T
0.5858028386650075	0.4325899921719158	0.1482857070923543	T	T	T
0.5749999720000005	0.1988699999999994	0.0080600000000004	F	F	F
0.0790000009999972	0.0729800009999977	0.0802899979999978	F	F	F
0.9250000279999995	0.1988699999999994	0.0080600000000004	F	F	F
0.4209999919999987	0.0729800009999977	0.0802899979999978	F	F	F
0.4353252567958272	0.2924793071664794	0.2269251304879892	T	T	T
0.9187390524569701	0.4361653556153329	0.1533392880395387	T	T	T
0.7473020836280174	0.0709400151464182	0.2089480022515397	T	T	T
0.7500000000000000	0.0665100009999975	0.0776999939999996	F	F	F
0.7500000000000000	0.3774399930000030	0.0028799999999976	F	F	F
0.2500000000000000	0.4419099979999999	0.0051800000000028	F	F	F
0.2456311559040352	0.4528426951049699	0.1552468567671461	T	T	T
0.2429001302013459	0.1487617585292845	0.2206018643321485	T	T	T
0.2500000000000000	0.2530299960000022	0.0756800030000022	F	F	F
0.7487516532608661	0.2476187643467096	0.1587917685272467	T	T	T
0.0769104295051817	0.8043689141686482	0.2053428867066228	T	T	T
0.5825472999683851	0.9335007149442189	0.1530570377038719	T	T	T
0.5749999720000005	0.6988699999999994	0.0080600000000004	F	F	F
0.0790000009999972	0.5729799639999982	0.0802899979999978	F	F	F
0.9250000279999995	0.6988699999999994	0.0080600000000004	F	F	F
0.4209999919999987	0.5729799639999982	0.0802899979999978	F	F	F
0.4243612699984349	0.8042312564974838	0.2041673624790768	T	T	T
0.9177371749225639	0.9337426763243647	0.1534942154020930	T	T	T
0.7241403804951694	0.5652962895833420	0.2184085849606033	T	T	T
0.7500000000000000	0.5665100379999970	0.0776999939999996	F	F	F
0.7500000000000000	0.8774399639999970	0.0028799999999976	F	F	F
0.2500000000000000	0.9419100269999987	0.0051800000000028	F	F	F
0.2500000000000000	0.9481999870000024	0.1594399929999994	F	F	F
0.2502128521869511	0.6366166766838348	0.2336345800400944	T	T	T
0.2497676304299158	0.7539120646259507	0.0687746716271450	T	T	T
0.7510756600233863	0.7448920708840164	0.1572319182917954	T	T	T

0.4059999979999986	0.4534200180000028	0.0846099999999979	F	F	F
0.9170000120000026	0.3119700080000030	0.0000000000000000	F	F	F
0.4190807638021111	0.4493866191872283	0.2101157977074583	T	T	T
0.9091708598193167	0.3178850836928872	0.1542619986107355	T	T	T
0.9103690161711456	0.0576262906939106	0.1443367618749920	T	T	T
0.4204939029340690	0.1873778551096452	0.2453703869298221	T	T	T
0.9059999979999986	0.0783400010000008	0.0109400010000016	F	F	F
0.4160000030000006	0.1855500019999994	0.0739599959999993	F	F	F
0.5883989300000001	0.0573568517036069	0.1439068099557318	T	T	T
0.0867349868599526	0.2092905355562567	0.2460812222132782	T	T	T
0.5940000020000014	0.0783400010000008	0.0109400010000016	F	F	F
0.0839999969999994	0.1855500019999994	0.0739599959999993	F	F	F
0.0939999949999972	0.4534200180000028	0.0846099999999979	F	F	F
0.5829999879999974	0.3119700080000030	0.0000000000000000	F	F	F
0.0903555720668763	0.4183892397443804	0.2147616420250107	T	T	T
0.5859924738121673	0.3184534495240371	0.1663812202804152	T	T	T
0.7500000000000000	0.4409900120000003	0.0794299969999983	F	F	F
0.7509152239287308	0.4528517544424133	0.2106834077694691	T	T	T
0.2451869939793462	0.0696100510985608	0.1555999453439467	T	T	T
0.2500000000000000	0.0603599989999992	0.0089200000000034	F	F	F
0.2500000000000000	0.3249400019999982	0.0025899999999979	F	F	F
0.2653228373806815	0.3044130963075861	0.1596356461325814	T	T	T
0.7522139372693650	0.1750584258843700	0.2251771729159604	T	T	T
0.7500000000000000	0.1905300040000029	0.0756800030000022	F	F	F
0.4059999979999986	0.9534199590000014	0.0846099999999979	F	F	F
0.9170000120000026	0.8119700080000030	0.0000000000000000	F	F	F
0.4150823137864986	0.9176192582630111	0.2153767540908167	T	T	T
0.9061163245478504	0.8177707359851412	0.1559695289716634	T	T	T
0.9092301332847592	0.5562507623737316	0.1445543228091206	T	T	T
0.4081001847081048	0.7080050443999263	0.2432052296954871	T	T	T
0.9059999979999986	0.5783400090000015	0.0109400010000016	F	F	F
0.4160000030000006	0.6855500019999994	0.0739599959999993	F	F	F
0.5895328855350884	0.5582401626047251	0.1427115140846424	T	T	T
0.0961480450287661	0.7090603811241608	0.2459857400580940	T	T	T
0.5940000020000014	0.5783400090000015	0.0109400010000016	F	F	F
0.0839999969999994	0.6855500019999994	0.0739599959999993	F	F	F
0.0939999949999972	0.9534199590000014	0.0846099999999979	F	F	F

0.5829999879999974	0.8119700080000030	0.0000000000000000	F	F	F
0.0854150346953258	0.9179986536000642	0.2158923502670791	T	T	T
0.5955403646413692	0.8177494066574129	0.1551697505223816	T	T	T
0.7500000000000000	0.9409899819999978	0.0794299969999983	F	F	F
0.7495362176650876	0.9594716422550870	0.2153895180923159	T	T	T
0.2460011978878071	0.5726380437724981	0.1523004566204922	T	T	T
0.2500000000000000	0.5603600319999984	0.0089200000000034	F	F	F
0.2500000000000000	0.8249400019999982	0.0025899999999979	F	F	F
0.2497810356872526	0.8092796530678446	0.1488608534755547	T	T	T
0.7514576980177602	0.6730622321347399	0.2244708009857445	T	T	T
0.7500000000000000	0.6905300190000006	0.0756800030000022	F	F	F
0.2687504778410943	0.5508121729381225	0.2940867880306491	T	T	T
0.3383850414521528	0.4648998200278759	0.4093219097788622	T	T	T
0.4207584046089450	0.3551208409114729	0.3051087822913017	T	T	T
0.5970191070875036	0.5383288601445894	0.2972107768112924	T	T	T
0.4972025175424281	0.4578869587042764	0.4118055102507450	T	T	T
0.4255554539907186	0.4727248757254520	0.3078722530994165	T	T	T

Table S3 The CONTCAR file of the most stable Pt on γ-Al_2O_3 surface

Al O Pt

32 48 1

Selective dynamics

Direct

0.0760727877603664	0.3043498930923090	0.2094908963255139	T	T	T
0.5854460036393672	0.4355840896005492	0.1520275817200898	T	T	T
0.5749999720000005	0.1988699999999994	0.0080600000000004	F	F	F
0.0790000009999972	0.0729800009999977	0.0802899979999978	F	F	F
0.9250000279999995	0.1988699999999994	0.0080600000000004	F	F	F
0.4209999919999987	0.0729800009999977	0.0802899979999978	F	F	F
0.4338462673428804	0.3097545717582723	0.2080594441360980	T	T	T
0.9203273181646421	0.4338844051540005	0.1531763477215278	T	T	T
0.7344022582494726	0.0711500518767793	0.2117745001347485	T	T	T
0.7500000000000000	0.0665100009999975	0.0776999939999996	F	F	F
0.7500000000000000	0.3774399930000030	0.0028799999999976	F	F	F
0.2500000000000000	0.4419099979999999	0.0051800000000028	F	F	F
0.2512591419123850	0.4483121768432692	0.1589241896833072	T	T	T
0.2517933963102448	0.1422990779652787	0.2291260937427934	T	T	T
0.2500000000000000	0.2530299960000022	0.0756800030000022	F	F	F
0.7549879596716728	0.2462177380234710	0.1566779929161251	T	T	T
0.0716813799105621	0.8061548910127528	0.2060806004332598	T	T	T
0.5821056602174978	0.9335620111398758	0.1517937318283457	T	T	T
0.5749999720000005	0.6988699999999994	0.0080600000000004	F	F	F
0.0790000009999972	0.5729799639999982	0.0802899979999978	F	F	F
0.9250000279999995	0.6988699999999994	0.0080600000000004	F	F	F
0.4209999919999987	0.5729799639999982	0.0802899979999978	F	F	F
0.4271186676526120	0.8053098261302530	0.2061287520910347	T	T	T
0.9160877969359995	0.9360347684470688	0.1535783217412243	T	T	T
0.7516387875493227	0.5710910803764756	0.2090340813333711	T	T	T
0.7500000000000000	0.5665100379999970	0.0776999939999996	F	F	F
0.7500000000000000	0.8774399639999970	0.0028799999999976	F	F	F
0.2500000000000000	0.9419100269999987	0.0051800000000028	F	F	F
0.2500000000000000	0.9481999870000024	0.1594399929999994	F	F	F
0.2493072974334106	0.6567465469544003	0.2179971650172015	T	T	T
0.2499323570543834	0.7550029461523154	0.0680076235242146	T	T	T
0.7499710162313605	0.7458773204380783	0.1579981034746644	T	T	T

0.4059999979999986	0.4534200180000028	0.0846099999999979	F	F	F
0.9170000120000026	0.3119700080000030	0.0000000000000000	F	F	F
0.4183246833513756	0.4207146631854091	0.2158749729174569	T	T	T
0.9117154820319325	0.3180406936428190	0.1558279636366870	T	T	T
0.9077286277293943	0.0570950130792068	0.1449302699593946	T	T	T
0.4188027357291717	0.2131329659349154	0.2491736209563935	T	T	T
0.9059999979999986	0.0783400010000008	0.0109400010000016	F	F	F
0.4160000030000006	0.1855500019999994	0.0739599959999993	F	F	F
0.5871135225341354	0.0574627578548231	0.1425101527164194	T	T	T
0.0974513598263779	0.2085598517827314	0.2483972145339264	T	T	T
0.5940000020000014	0.0783400010000008	0.0109400010000016	F	F	F
0.0839999969999994	0.1855500019999994	0.0739599959999993	F	F	F
0.0939999949999972	0.4534200180000028	0.0846099999999979	F	F	F
0.5829999879999974	0.3119700080000030	0.0000000000000000	F	F	F
0.0884939333064828	0.4179402874397669	0.2162742940544907	T	T	T
0.5989363417440574	0.3192545743210373	0.1558541561335772	T	T	T
0.7500000000000000	0.4409900120000003	0.0794299969999983	F	F	F
0.7523449036130211	0.4593787046027895	0.2154148860178491	T	T	T
0.2474292054834402	0.0709994574742355	0.1557188808217468	T	T	T
0.2500000000000000	0.0603599989999992	0.0089200000000034	F	F	F
0.2500000000000000	0.3249400019999982	0.0025899999999979	F	F	F
0.2560277437005830	0.3049916415880436	0.1558135471249000	T	T	T
0.7569250533919031	0.1764946035122096	0.2243569292668699	T	T	T
0.7500000000000000	0.1905300040000029	0.0756800030000022	F	F	F
0.4059999979999986	0.9534199590000014	0.0846099999999979	F	F	F
0.9170000120000026	0.8119700080000030	0.0000000000000000	F	F	F
0.4145253069819413	0.9178101731847453	0.2157136590873367	T	T	T
0.9038770345642945	0.8195145332242512	0.1552490051426820	T	T	T
0.9119595808263389	0.5571842367671206	0.1441443027233482	T	T	T
0.4145260596105260	0.7090741412926629	0.2450429469728373	T	T	T
0.9059999979999986	0.5783400090000015	0.0109400010000016	F	F	F
0.4160000030000006	0.6855500019999994	0.0739599959999993	F	F	F
0.5896465439068612	0.5571773106314453	0.1442239697518423	T	T	T
0.0843118405505444	0.7095486241237409	0.2446190435078900	T	T	T
0.5940000020000014	0.5783400090000015	0.0109400010000016	F	F	F
0.0839999969999994	0.6855500019999994	0.0739599959999993	F	F	F
0.0939999949999972	0.9534199590000014	0.0846099999999979	F	F	F

0.5829999879999974	0.8119700080000030	0.0000000000000000	F	F	F
0.0852827848103603	0.9185440132698990	0.2158553803477995	T	T	T
0.5934890881010704	0.8180670179377603	0.1542279112545835	T	T	T
0.7500000000000000	0.9409899819999978	0.0794299969999983	F	F	F
0.7461938049858182	0.9586436057840733	0.2152638105744433	T	T	T
0.2502965160273957	0.5714882635022047	0.1567877459252483	T	T	T
0.2500000000000000	0.5603600319999984	0.0089200000000034	F	F	F
0.2500000000000000	0.8249400019999982	0.0025899999999979	F	F	F
0.2495417411553428	0.8049411796389246	0.1508784590949802	T	T	T
0.7499769436965136	0.6745780808479442	0.2250148805762740	T	T	T
0.7500000000000000	0.6905300190000006	0.0756800030000022	F	F	F
0.4866070195623783	0.1102270975859740	0.2965221267008927	T	T	T

Table S4 The CONTCAR file of the most stable Pt on WO_5/γ-Al_2O_3 surface

Al O W Pt

32 53 1 1

Selective dynamics

Direct

0.0808957382330410	0.3054713256539401	0.2084786899282108	T	T	T
0.5842677996234183	0.4313670742255885	0.1482638893296252	T	T	T
0.5749999720000005	0.1988699999999994	0.0080600000000004	F	F	F
0.0790000009999972	0.0729800009999977	0.0802899979999978	F	F	F
0.9250000279999995	0.1988699999999994	0.0080600000000004	F	F	F
0.4209999919999987	0.0729800009999977	0.0802899979999978	F	F	F
0.4457655910993520	0.2889217752637130	0.2251646304940924	T	T	T
0.9228102776422143	0.4305741511611322	0.1494139881044452	T	T	T
0.7475914200524704	0.0718818378062124	0.2091949745674312	T	T	T
0.7500000000000000	0.0665100009999975	0.0776999939999996	F	F	F
0.7500000000000000	0.3774399930000030	0.0028799999999976	F	F	F
0.2500000000000000	0.4419099979999999	0.0051800000000028	F	F	F
0.2499846350779787	0.4523962285968903	0.1567796523588105	T	T	T
0.2440919620233425	0.1483941719715059	0.2213956517316096	T	T	T
0.2500000000000000	0.2530299960000022	0.0756800030000022	F	F	F
0.7538548038820365	0.2468324872425803	0.1579377673488209	T	T	T
0.0703122838036947	0.8027994052974744	0.2043262271164822	T	T	T
0.5837444670680083	0.9353876789387852	0.1524870434425294	T	T	T
0.5749999720000005	0.6988699999999994	0.0080600000000004	F	F	F
0.0790000009999972	0.5729799639999982	0.0802899979999978	F	F	F
0.9250000279999995	0.6988699999999994	0.0080600000000004	F	F	F
0.4209999919999987	0.5729799639999982	0.0802899979999978	F	F	F
0.4178177280451826	0.8116799860814113	0.2042915180025323	T	T	T
0.9169328463038430	0.9341448258435274	0.1533493334925858	T	T	T
0.7464118751557610	0.5561231048928806	0.2149424270192088	T	T	T
0.7500000000000000	0.5665100379999970	0.0776999939999996	F	F	F
0.7500000000000000	0.8774399639999970	0.0028799999999976	F	F	F
0.2500000000000000	0.9419100269999987	0.0051800000000028	F	F	F
0.2500000000000000	0.9481999870000024	0.1594399929999994	F	F	F
0.2286622087331860	0.6390604721116888	0.2315224126989245	T	T	T
0.2495668419520450	0.7542264308888299	0.0679062171432004	T	T	T
0.7454100036780549	0.7433873929694080	0.1583754729599609	T	T	T

0.4059999979999986	0.4534200180000028	0.0846099999999979	F	F	F
0.9170000120000026	0.3119700080000030	0.0000000000000000	F	F	F
0.4215400551944840	0.4513426580299846	0.2123024404821692	T	T	T
0.9179173026838593	0.3147940901376176	0.1523537702516631	T	T	T
0.9103109205230611	0.0571677879147622	0.1444145744737317	T	T	T
0.4231567258820337	0.1852810511501488	0.2456070661600169	T	T	T
0.9059999979999986	0.0783400010000008	0.0109400010000016	F	F	F
0.4160000030000006	0.1855500019999994	0.0739599959999993	F	F	F
0.5886508346469185	0.0573267650327151	0.1438839758766391	T	T	T
0.0899424974014779	0.2093247935494270	0.2480665427675153	T	T	T
0.5940000020000014	0.0783400010000008	0.0109400010000016	F	F	F
0.0839999969999994	0.1855500019999994	0.0739599959999993	F	F	F
0.0939999949999972	0.4534200180000028	0.0846099999999979	F	F	F
0.5829999879999974	0.3119700080000030	0.0000000000000000	F	F	F
0.0922357285094116	0.4182615380541279	0.2149769351467983	T	T	T
0.5896739582606821	0.3169894135350874	0.1632320710654644	T	T	T
0.7500000000000000	0.4409900120000003	0.0794299969999983	F	F	F
0.7572876528647200	0.4455332731374939	0.2072600559242946	T	T	T
0.2445649576718732	0.0693140725491000	0.1559755110252711	T	T	T
0.2500000000000000	0.0603599989999992	0.0089200000000034	F	F	F
0.2500000000000000	0.3249400019999982	0.0025899999999979	F	F	F
0.2697482594124783	0.3039096104635778	0.1608222928101905	T	T	T
0.7542200034161773	0.1756716339631025	0.2248129632189974	T	T	T
0.7500000000000000	0.1905300040000029	0.0756800030000022	F	F	F
0.4059999979999986	0.9534199590000014	0.0846099999999979	F	F	F
0.9170000120000026	0.8119700080000030	0.0000000000000000	F	F	F
0.4147571248679444	0.9217611974551083	0.2156086288587958	T	T	T
0.8982616735086687	0.8173672262068997	0.1563331049105793	T	T	T
0.9090411302435313	0.5577291188780140	0.1464546055781357	T	T	T
0.3799226416667711	0.7212204713037109	0.2493886741108351	T	T	T
0.9059999979999986	0.5783400090000015	0.0109400010000016	F	F	F
0.4160000030000006	0.6855500019999994	0.0739599959999993	F	F	F
0.5942597301447405	0.5561012550096897	0.1421787082624971	T	T	T
0.0686061980454837	0.7048467076385319	0.2400925391458522	T	T	T
0.5940000020000014	0.5783400090000015	0.0109400010000016	F	F	F
0.0839999969999994	0.6855500019999994	0.0739599959999993	F	F	F
0.0939999949999972	0.9534199590000014	0.0846099999999979	F	F	F

0.5829999879999974	0.8119700080000030	0.0000000000000000	F	F	F
0.0844375206214807	0.9161413803448583	0.2154886861438874	T	T	T
0.5916664573813876	0.8171095196729994	0.1576018115773075	T	T	T
0.7500000000000000	0.9409899819999978	0.0794299969999983	F	F	F
0.7490201976525204	0.9598556520628057	0.2155187204745598	T	T	T
0.2425721953225036	0.5732445342648602	0.1531211466692454	T	T	T
0.2500000000000000	0.5603600319999984	0.0089200000000034	F	F	F
0.2500000000000000	0.8249400019999982	0.0025899999999979	F	F	F
0.2466816223540223	0.8088584127736521	0.1490541398024108	T	T	T
0.7278985741031203	0.6693477726878285	0.2294734517417060	T	T	T
0.7500000000000000	0.6905300190000006	0.0756800030000022	F	F	F
0.3069479866472225	0.5690350450610695	0.2994006124960933	T	T	T
0.3335467900532402	0.4516321933021371	0.4002523145678076	T	T	T
0.4628335520191705	0.3533882741923141	0.3030629412998135	T	T	T
0.6487908278392427	0.5287638042118050	0.3046278213582810	T	T	T
0.5009257894451398	0.4770996366845351	0.4108154034659010	T	T	T
0.4448162127097792	0.4669147175031895	0.3147461658048205	T	T	T
0.5214549850619212	0.6299969833464739	0.2797482780441120	T	T	T

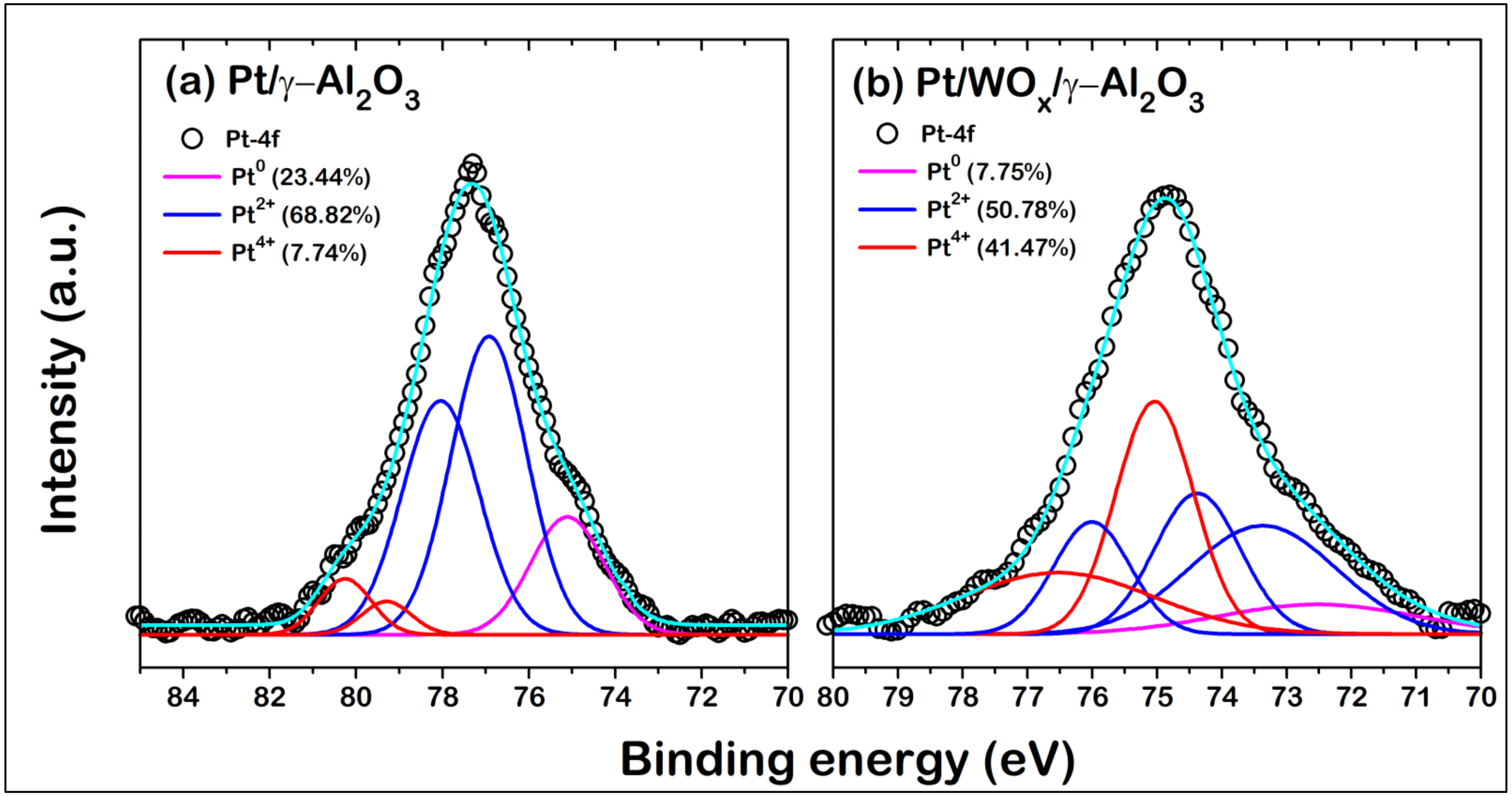

Figure **S1** The XPS of Pt4f electrons from the Pt/γ-Al_2O_3 and Pt/WOx/γ-Al_2O_3
